# C-reactive protein (CRP) but not the related pentraxins serum amyloid P and PTX3 inhibits the proliferation and induces apoptosis of the leukemia cell line Mono Mac 6

**DOI:** 10.1186/s12865-017-0230-z

**Published:** 2017-12-04

**Authors:** Wensheng Chen, Darrell Pilling, Richard H. Gomer

**Affiliations:** 1 Department of Biology, Texas A&M University, College Station, TX 77843-3474 USA; 2 Institute of Clinical Pharmacology, Key Laboratory of Anti-inflammatory and Immune Medicine, Ministry of Education, Anhui Collaborative Innovation Center of Anti-inflammatory and Immune Medicine, Anhui Medical University, Hefei, 230032 China

**Keywords:** C reactive protein, Pentraxin, Leukemia, Apoptosis

## Abstract

**Background:**

Pentraxins are a family of highly conserved secreted proteins that regulate the innate immune system, including monocytes and macrophages. C-reactive protein (CRP) is a plasma protein whose levels can rise to 1000 μg/ml from the normal <3 μg/ ml during inflammation.

**Results:**

We find that CRP inhibits proliferation of the human myeloid leukemia cell line Mono Mac 6 with an IC50 of 75 μg/ ml by inducing apoptosis of these cells. The related proteins serum amyloid P (SAP) and pentraxin 3 (PTX3) do not inhibit Mono Mac 6 proliferation. CRP has no significant effect on the proliferation of other leukemia cell lines such as HL-60, Mono Mac 1, K562, U937, or THP-1, or the survival of normal peripheral blood cells. The effect of CRP appears to be dependent on the CRP receptor FcγRI, and is negatively regulated by a phosphatidylinositol −3-kinase pathway.

**Conclusion:**

These data reveal differential signaling by pentraxins on immune cells, and suggest that CRP can regulate the proliferation of some myeloid leukemia cells.

## Background

Pentraxins are a family of highly conserved secreted proteins that regulate the innate immune system, including cells of myeloid lineage such as neutrophils, monocytes, and macrophages [1–3]. In healthy humans, the plasma levels of CRP and PTX3 are low (< 2 μg/ml and <25 ng/ml respectively) and SAP is approximately 30 μg/ml, whereas during inflammation CRP and PTX3 levels may rise to 50–500 μg/ml and 200–800 ng/ml respectively, but SAP levels remain constant [2]. Serum amyloid P (SAP) reduces neutrophil activation and recruitment to sites of inflammation, regulates monocyte differentiation, and inhibits the differentiation of monocytes into fibroblast-like cells called fibrocytes [4–7]. In animal models and in human clinical trials, injections of SAP decrease fibrosis, indicating that SAP has a dominant effect on a disease that is mediated in part by the innate immune system [3, 8, 9]. CRP is pro-inflammatory and promotes fibrosis [10, 11]. However, under some conditions, CRP decreases inflammation, indicating that much remains to be understood about this molecule [12–14]. PTX3 is associated with inflammation in humans, but in mice appears to be pro-inflammatory in some models and limits inflammation in other models [15, 16]. SAP, CRP, and PTX3 all bind to multiple receptors including IgG Fcγ receptors (FcγR; CD16, CD32a, and CD64), the IgA receptor (FcαR; CD89), P-selectin (CD62P), L-selectin (CD62L), and the lectin dendritic cell-specific intercellular adhesion molecule-3-grabbing non-integrin (DC-SIGN/ CD209) [5, 17–21]. These receptors are expressed on a variety of immune cells including monocytes, mature tissue macrophages, and also cell lines derived from monocyte lineage leukemias [22]. However, our understanding of the effects on cells of pentraxins binding to these receptors is still incomplete [17–19].

SAP and PTX3 can affect tumors, either by regulating cancer-related inflammation, angiogenesis, or directly inhibiting cancer cell growth and differentiation [9, 23–26]. However, the role of CRP in less well understood. Elevated serum CRP levels are associated with poor prognosis in solid tumors, probably as an indicator of chronic inflammation associated with tumor progression, but the role of CRP in leukemia is unclear [27, 28].

In this report, we find that CRP inhibits the proliferation, and induces apoptosis of, Mono Mac 6 cells, but has no effect on the survival of normal peripheral blood cells. The effect of CRP on Mono Mac 6 cells appears to be dependent on CD64 (FcγRI) and the IgA receptor (FcαR; CD89), and is negatively regulated by a phosphatidylinositol-3-kinase (PI3K) dependent pathway. These data reveal differential signaling by pentraxins on immune cells, and suggest that CRP may be a novel regulator of some subtypes of leukemia.

## Methods

### Human PBMC isolation and culture, and leukemia cell culture

Human peripheral blood was collected into heparin tubes (Greiner Bio-One, Monroe, NC) from healthy adult volunteers who gave written consent and with specific approval from the Texas A&M University human subjects Institutional Review Board. Peripheral blood mononuclear cells (PBMC) were isolated from the blood using Ficoll-Paque Plus (GE Healthcare Biosciences, Piscataway, NJ), as described previously [4, 29]. HL-60, K562, THP-1, U937 (ATCC, Manassas, VA), Mono Mac 1 and Mono Mac 6 (DSMZ, Braunschweig, Germany) were grown in RPMI 1640 with 10% bovine calf serum (BCS) (VWR-Seradigm, Radnor, PA) containing 2 mM glutamine, 100 U/ml penicillin, and 100 μg/ml streptomycin (all from Lonza, Walkersville, MD). K562 is a chronic myeloid leukemia cell line [30], U937 is a lymphoma cell line [31], and HL-60 [32], THP-1 [33], Mono Mac 1, and Mono Mac 6 [34], are acute myeloid leukemia cell lines. Each individual experiment was performed in duplicate or triplicate, and repeated at least three separate times. For experiments involving human peripheral blood, at least three different donors were used for each experiment. Data are presented as the mean and standard error of the mean (SEM).

### Proliferation assay

Purified human SAP (EMD, Billerica, MA), purified human CRP (Fitzgerald Industries, Acton, MA) and mammalian NSO cell-derived recombinant human PTX3 (R&D Systems, Minneapolis, MN) were diluted with RPMI culture medium. As commercial SAP preparations contain 0.1% azide, we buffer-exchange the SAP into 20 mM sodium phosphate, pH 7.4, as described previously [35]. Leukemia cell lines were grown on 6-well plates (353,046; Falcon, Corning Inc., Corning, NY) until 80–90% confluence. Cells were subcultured at a ratio of 1:5, and cells from the second passage were used in the proliferation assay. Cell lines (2 × 10^4^/well) and PBMCs (2 × 10^5^/well), in the presence or absence of pentraxins, were plated in 96-well plates (353,072; Falcon) in 200 μl RPMI 1640 containing 5% BCS. The inhibitors PP2, PP3, U0126, Wortmannin, H-89, and okadaic acid were from EMD. LY294002 and the Syk inhibitor were from BioVision (Milpitas, CA). NSC23766, ML141, and Ras inhibitory peptide were from Cayman Chemicals (Ann Arbor, MI). SP600125 and an aqueous solution of 100 mM sodium vanadate were from NEB (Ipswich, MA). Inhibitors, apart from vanadate, were dissolved in DMSO at 10–100 mM, and diluted to final concentrations in RPMI culture medium. At 48 h, the cells were resuspended, stained with 5 μg/ml propidium iodide (Thermo Rockford, IL) following [36] and then counted using an Accuri C6 flow cytometer (BD-Biosciences, San Jose, CA).

### Immunohistochemistry and flow cytometry

Mono Mac 6 cells were cultured with CRP for 48 h and then fixed and stained as described previously [7]. Mono Mac 6 cells were stained with rabbit monoclonal antibodies to KI-67 (Novus, Littleton, CO) or cleaved Caspase3 (R&D Systems, Minneapolis, MN). Isotype-matched irrelevant rabbit polyclonal antibodies (Bethyl Laboratories, Montgomery, TX), were used as a control. Secondary F(ab’)2 biotin-conjugated donkey anti-rabbit antibodies were from Jackson ImmunoResearch (West Grove, PA). Staining was revealed with streptavidin-alkaline phosphatase (Invitrogen, Grand Island, NY) and Vector Red Alkaline Phosphatase Kit (Vector Laboratories, Burlingame, CA) following the manufacturers’ instructions, and slides were then counterstained with hematoxylin. For flow cytometry, PBMCs and leukemia cells were cultured for 48 h in the presence or absence of pentraxins and stained as previously described [5]. PBMCs were stained with mouse monoclonal antibodies to CD14 (clone 63D3, isotype: mouse IgG1, BioLegend, San Diego, CA), or CD16 (clone GRM1, mouse IgG2a, Southern Biotech, Birmingham, AL). Leukemia cells were stained with mouse monoclonal antibodies to CD16 (clone GRM1, mouse IgG2a, Southern Biotech) CD32a (clone IV3, mouse IgG2b, Stem Cell, Cambridge, MA), pan CD32 (clone Fun2, mouse IgG2b, BioLegend), CD64 (clone 10.1, mouse IgG1, BioLegend), CD89 (clone A59, mouse IgG1, BioLegend), or CD209 (clone 9E9A8, mouse IgG2a, BioLegend). Isotype-matched irrelevant mouse monoclonal antibodies (BioLegend) were used as controls. Alexa Fluor 488-conjugated F(ab)‘2 Donkey anti mouse IgG antibody were from Jackson ImmunoResearch. Staining was measured by flow cytometry.

### Western blot analysis

For cell extracts, cells were lysed in RIPA buffer (Thermo) containing protein and phosphatase inhibitor cocktail (Cell Signaling Technologies, Danvers, MA) for 30 min on ice. Samples were clarified by centrifugation at 14,000 x g for 20 min at 4 °C. Supernatants were collected, mixed with Laemmli sample buffer, separated on 4–20% TRIS-glycine gels (Lonza), and transferred to PVDF membrane (EMD) and blocked, as described previously [4, 37]. Membranes were incubated overnight at 4 °C with a 1/1000 dilution of rabbit antibodies against active caspase 3, phosphorylated ERK, PTEN (Cell Signaling Technology) or 500 ng/ml anti-mouse actin (Genscript, Piscataway, NJ). Membranes were washed in TBS/ 0.1% (*v*/v) Tween 20 (Thermo). Antibodies were detected with 1 μg/ml peroxidase conjugated goat Fab_2_ anti-rabbit or anti-mouse antibodies (Jackson ImmunoResearch), as described previously [4]. SuperSignal West Pico Chemiluminescence Substrate (Thermo) was used following the manufacturer’s protocol to visualize the peroxidase using a ChemiDoc XRS+ System (BioRad Hercules, CA).

### Blocking assays

Mouse monoclonal antibodies to CD16 (GRM1 and 3G8, mouse IgG1 BioLegend), CD32 (Fun2; AT10 mouse IgG1, BioRad; and 6C4, mouse IgG1, eBioscience, Rockford, IL), CD64 (clone 10.1), and CD89 (clone A59), mouse IgG1, IgG2a, and IgG2b (all from BioLegend), and human Fc blocker (BD Biosciences) or human Fc receptor binding inhibitor (eBioscience) diluted to a final concentration of 10 μg/ml were added to Mono Mac 6 cells in the presence or absence of CRP. At 48 h, the cells were resuspended and stained with 5 μg/ml propidium iodide and analyzed by flow cytometry. CRP and mouse monoclonal antibodies to CD32 (clone Fun2), CD64 (clone 10.1), or CD89 (clone A59) were also added to Mono Mac 6 cells in the presence or absence of 10 μg/ml donkey anti-mouse antibodies (Jackson ImmunoResearch) to cross-link the antibodies. At 48 h, the cells were resuspended and stained with 5 μg/ml propidium iodide and analyzed by flow cytometry.

### Cell cycle assay

Mono Mac 6 cells were cultured with or without 75 μg/ml CRP for 48 h. Cells were then collected by centrifugation and resuspended in, and permeabilized with, ice cold 70% ethanol, and then stored at −20 °C for 24 h. After permeabilization, cells were collected by centrifugation, resuspended in PBS, and stained with 5 μg/ml 7-AAD (Ana Spec, Fremont, CA) and analyzed by flow cytometry [36].

### Statistics

Statistical analysis was performed using Prism (GraphPad, San Diego, CA). Differences between two groups were assessed by t test, or between multiple groups using analysis of variance (ANOVA) with Dunnett’s post-test. Significance was defined as *p* < 0.05.

## Results

### CRP inhibits the proliferation of mono Mac 6 cells

**Fig. 1:**
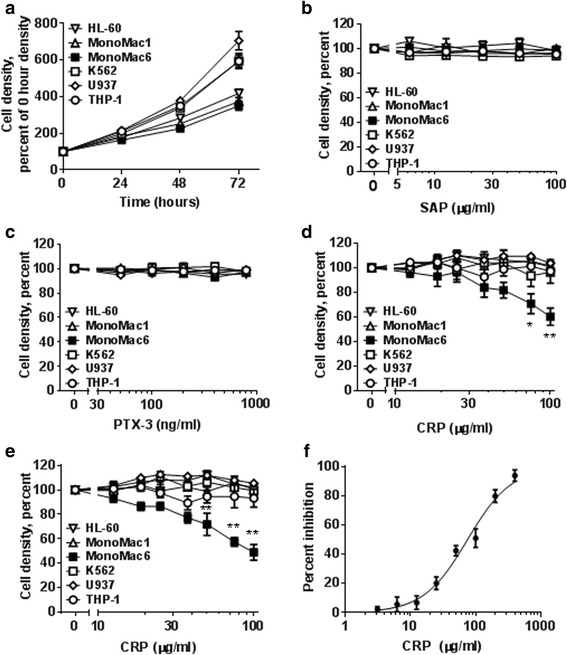
CRP inhibits the proliferation of Mono Mac 6 cells. **a** Increase in cell density of 6 leukemia cell lines cultured without pentraxins at 0, 24, 48, and 72 h. The cell density at 0 h was 1 × 10^5^/ml. **b** Cell densities of 6 leukemia cell lines cultured with SAP at 48 h as a percent of the 48 h 0 SAP density. **c** Cell densities of 6 leukemia cell lines cultured with PTX3 at 48 h as a percent of the 48 h 0 PTX3 density. **d** Cell densities of 6 leukemia cell lines cultured with CRP at 24 h as a percent of the 24 h 0 CRP density. **e** Cell densities of 6 leukemia cell lines cultured with CRP at 48 h as a percent of the 48 h 0 CRP density. **f** Inhibition of Mono Mac 6 proliferation by CRP at 48 h, with 0 inhibition indicating proliferation corresponding to no CRP, and 100% inhibition corresponding to a density equal to the starting cell density. Data were fit to sigmoidal dose–response curves with a variable Hill coefficient. Values are mean ± SEM, *n* = 3 independent experiments. In **d** and **e**, * indicates *p* < 0.05 and ** indicates *p* < 0.01 compared to 0 μg/ml CRP (t-test)

Since pentraxin receptors are expressed on monocytes, an intriguing possibility is that pentraxins might affect the proliferation or viability of some monocyte-derived leukemia cell lines. To test this, we added pentraxins to the monocyte-derived leukemia cell lines HL-60, Mono Mac 1, Mono Mac 6, K562, U937, and THP-1. In the absence of added pentraxins, all five cell lines proliferated as previously reported [30–34] (Fig. 1a). 6.25 to 100 μg/ml SAP, encompassing the ~30 μg/ ml human SAP serum concentration [38], and 50 to 800 ng/ml PTX3, encompassing the range in human serum during inflammation [39], did not show a significant effect on the proliferation of the cells over the course of 48 h (Fig. 1b and c). However, 50 to 100 μg/ml CRP decreased the numbers of Mono Mac 6 cells at 24 and 48 h (Fig. 1d, e). CRP levels in the plasma of healthy adults are ≤2 μg/ml, but increase up to ~1000 μg/ml following an acute phase stimulus [14, 40]. The half maximal inhibitory concentration (IC50) of CRP on Mono Mac 6 was 76 ± 4 μg /ml, with a Hill coefficient of 1.3 ± 0.1 (Fig. 1f). Together, these data suggest that exogenous CRP can decrease the number of Mono Mac 6 cells in culture.

### CRP does not significantly affect PBMC viability

**Fig. 2:**
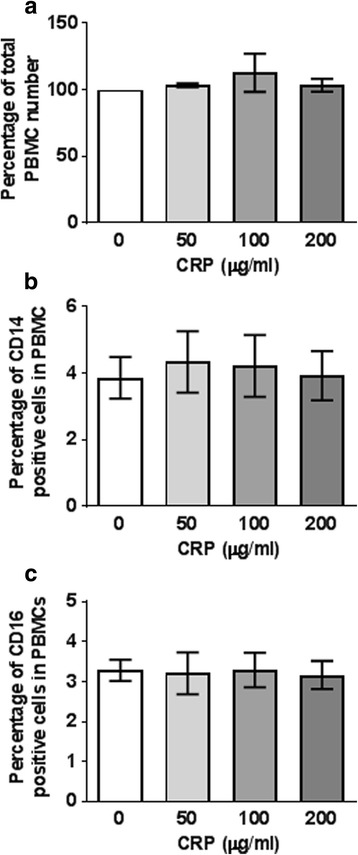
CRP does not affect PBMC viability. **a** The number of total PBMC after incubation with or without CRP for 48 h as a percent of the number at 48 h with no CRP. **b** The percent of CD14^+^ cells in the cultures in A. **c** The percent of CD16^+^ cells in the cultures in A. Values are mean ± SEM, n = 3 independent experiments, with each experiment using PBMC from a different donor

To determine if CRP affects normal cells in addition to Mono Mac 6 cells, we added CRP to freshly isolated PBMCs from healthy donors and counted total viable cells. After 48 h, there was no significant effect on cell numbers (Fig. 2a). Mono Mac 6 cells express phenotypic and functional features of mature monocytes [41], and mature monocytes express CD14, with a subset additionally expressing CD16 [42, 43]. 50 to 200 μg/ml CRP did not significantly affect the number of CD14+ or the number of CD16+ cells in the cultured PBMCs (Fig. 2b and c). Together, these results suggest that the ability of CRP to decrease the proliferation of Mono Mac 6 cells is not due to a general effect on monocyte viability.

### Mono Mac 6 cells do not express a unique CRP receptor

**Fig. 3:**
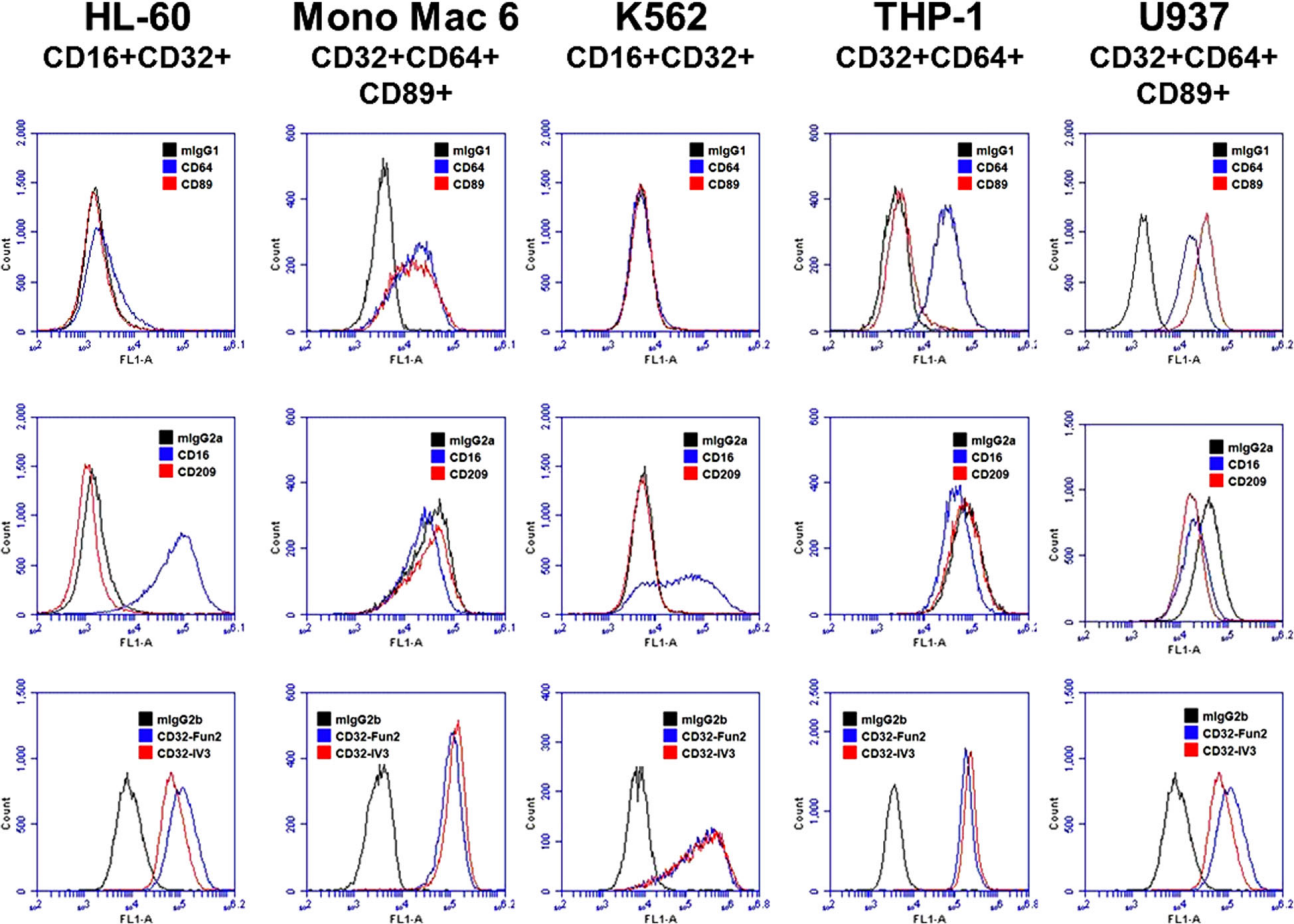
CRP receptor expression on 5 leukemia cell lines. CD16, CD32, CD64, CD89, and CD209 expression on the indicated cell lines was examined by flow cytometry. Top row: Blue trace is CD64, red is CD89. Middle row: Blue is CD16, red is CD209. Bottom row: Blue is CD32-Fun2, Red is CD32-IV3. Black traces are control IgGs. Plots are representative of 3 independent experiments

CRP interacts with the innate immune system through its binding to receptors such as CD16, CD32a, CD64, and CD89 [17, 18, 21]. To test whether the inhibitory effect of CRP on Mono Mac 6 cells is due to these cells expressing a known CRP receptor that is not expressed on the CRP-insensitive cells, we examined known CRP receptors on HL-60, Mono Mac 6, K562, THP-1, and U937 cells. Mono Mac 6 expressed detectable CD32, CD64, and CD89 (Fig. 3), but those three receptors were also detected on at least one of the CRP-insensitive cell lines. U937 expressed detectable CD32, CD64, and CD89. THP-1 expressed CD32 and CD64. HL-60 and K562 expressed detectable CD16 and CD32 but not CD64 or CD89. All the cell lines appear to be CD32a expressing cells, as they all bound the CD32 clone IV.3 [44], and the detection of CD64 by Mono Mac 6, THP-1, and U937 cells correlated with high binding of the mouse IgG2ba isotype, which binds through its Fc domain to CD64 [45]. None of the cell lines expressed detectable CD209. These data indicate that the CRP effect on Mono Mac 6 cells may be due to a unique combination of CRP receptors, an unknown CRP receptor, or a proliferation-inhibiting signal transduction pathway that is present in Mono Mac 6 cells but is not functional in the four CRP-insensitive cell lines.

### Anti-CD64 antibodies attenuate the CRP effect on mono Mac 6 cells

**Fig. 4:**
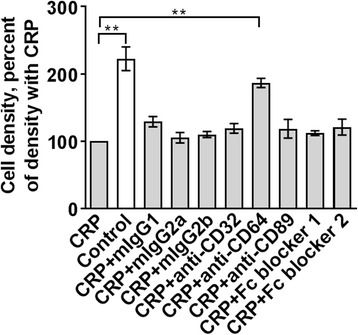
Anti-CD64 antibodies attenuate the CRP effect on Mono Mac 6 cells. Anti-CD32, anti-CD64, anti-CD89 antibodies, mouse IgG isotypes, or 2 Fc blockers were added to Mono Mac 6 cells for 2 h before 75 μg/ml CRP was added to the cells. Cell densities at 48 h were normalized to the density with CRP alone. Values are mean ± SEM, *n* = 3 independent experiments. ** indicates *p* < 0.01 (t-test)

In some cases, isotype IgG or anti-receptor antibodies can block the binding of a ligand to the receptor and prevent receptor activation by the ligand [45, 46]. To determine if the CRP effect on Mono Mac 6 cells can be similarly blocked with antibodies against a known CRP receptor, anti-CD16, anti-CD32, anti-CD64, anti-CD89 antibodies, isotype IgGs, or 2 different Fc blockers (IgG Fc fragments) were added to Mono Mac 6 cells for 2 h before 75 μg/ml CRP was added to the cells. Cells were counted after 48 h. CRP inhibited the proliferation of Mono Mac 6 as previously described (Fig. 4). Anti-CD64 antibodies attenuated this inhibition, while the other reagents did not show a significant effect (Fig. 4). As mouse IgG2a, which binds with high affinity to the IgG binding site of CD64 [45] and the Fc blockers did not attenuate the CRP effect, these data suggests that CRP may bind to CD64 at a site distinct from the IgG binding site, and the binding of anti-CD64 antibodies either directly or allosterically inhibits the CRP effect on CD64. These data suggest that CD64 plays a role in, or affects, the CRP proliferation-inhibiting signal transduction pathway in Mono Mac 6 cells.

### CRP regulates FcR expression on mono Mac 6

**Fig. 5:**
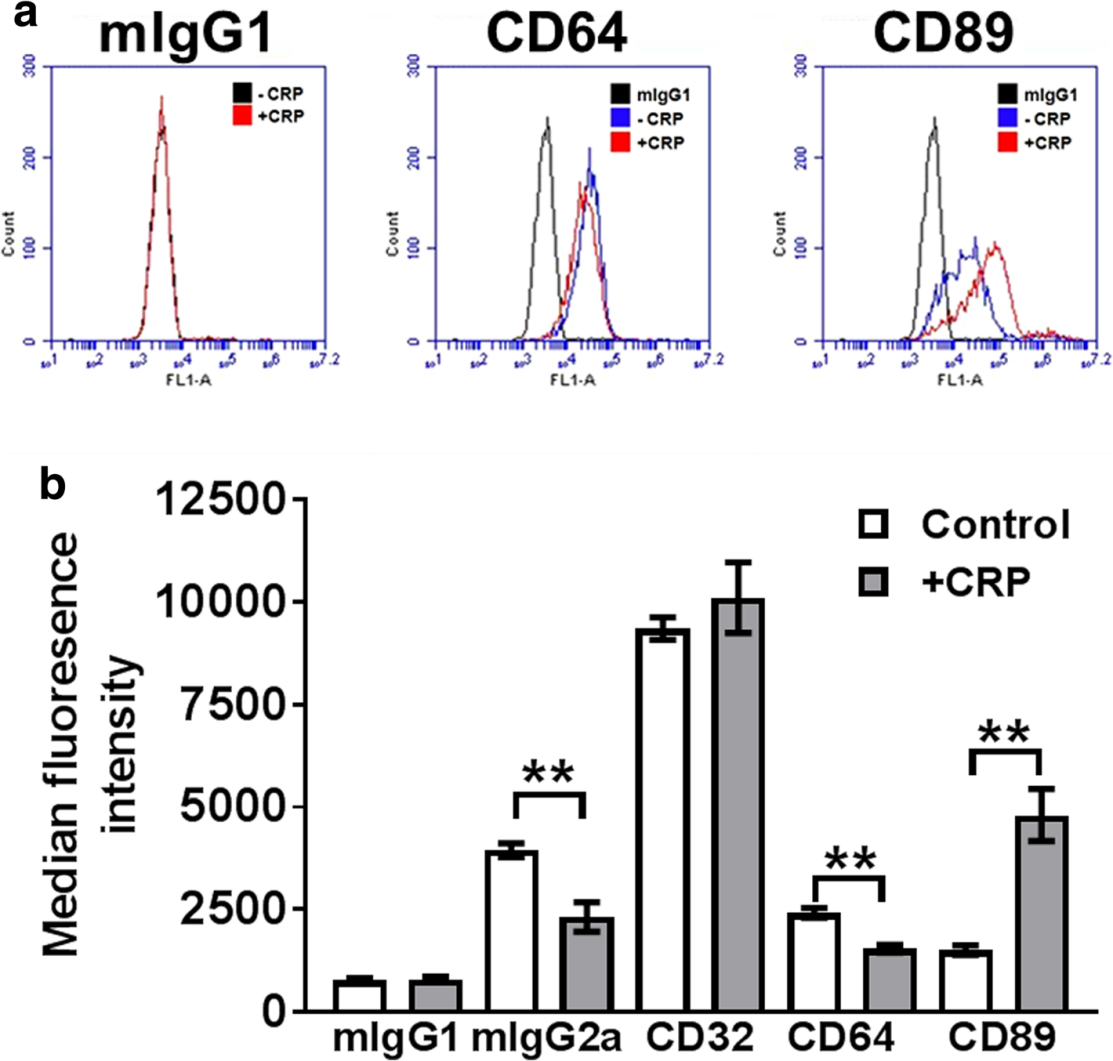
CRP regulates receptor expression on Mono Mac 6 cells. **a** Flow cytometry analysis of CD64 and CD89 expression on Mono Mac 6 cells in the presence or absence of CRP at 48 h. **b** Quantification of mouse IgG isotype, CD32, CD64, and CD89 antibody binding to Mono Mac 6 cells after incubation with or without CRP for 48 h. Values are mean ± SEM, *n* = 4 independent experiments. ** indicates p < 0.01 (t-test)

To determine if CRP affects FcR expression on Mono Mac 6 cells, 75 μg/ml CRP was added to Mono Mac 6 cells and CD32, CD64, and CD89 expression was examined after 48 h. CRP had no significant effect on CD32 expression, increased CD89 expression, and decreased CD64 expression (Fig. 5). These results indicate that CRP increased levels of CD89 on Mono Mac 6 cells, and possibly by conventional receptor downregulation after ligand binding [22], decreased levels of CD64. Another possibility is that the CRP, by binding to CD64, blocked the binding of the anti-CD64 antibodies.

### Cross-linking of FcRs does not mimic the CRP effect on mono Mac 6 cells

**Fig. 6:**
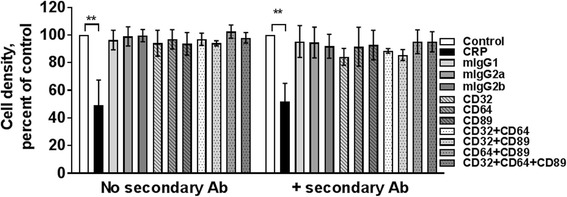
Cross-linking of FcRs does not mimic the CRP effect on Mono Mac 6 cells. CRP or the indicated mouse antibodies were added to Mono Mac 6 cells in the presence or absence of donkey Fab_2_ anti-mouse secondary antibodies to cross-link the mouse antibodies. Cells were counted after 48 h. Values are mean ± SEM, n = 3 independent experiments. ** indicates p < 0.01 (t-test)

Cross-linking of FcRs can lead to activation of downstream kinases such as spleen tyrosine kinase (Syk) and ERK [47]. To determine if receptor cross-linking can inhibit Mono Mac 6 proliferation, we added mouse pan anti-CD32 (Fun2), mouse anti-CD64, or mouse anti-CD89 antibodies to Mono Mac 6 cells, and then added donkey (Fab_2_) anti-mouse antibodies to cross-link the mouse antibodies and counted viable cell numbers after 48 h. CRP inhibited the proliferation of Mono Mac 6 cells (Fig. 6). The anti-CD32, anti-CD64, and anti-CD89 antibodies (alone or in combination, with or without secondary antibodies) did not significantly affect Mono Mac 6 proliferation (Fig. 6). Together, these results indicate that although anti-CD64 antibodies can partially block the ability of CRP to inhibit Mono Mac 6 proliferation, they cannot mimic the effect of CRP.

### PI3 kinase appears to inhibit the ability of CRP to regulate proliferation

**Fig. 7:**
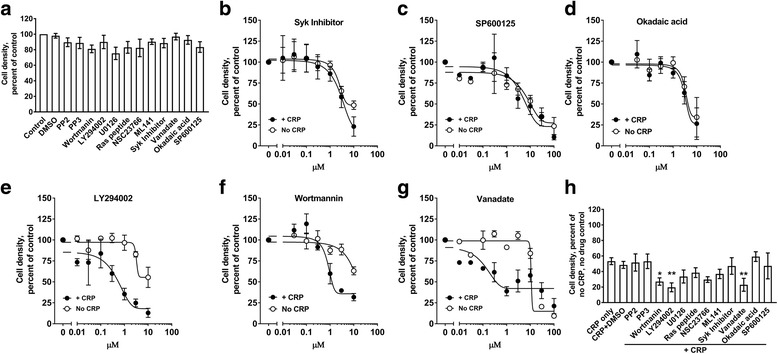
CRP is negatively regulated by a phosphatidylinositol-3-kinase (PI3K) dependent pathway. **a** Mono Mac 6 cells were incubated in the absence (control), 1/10,000 dilution of DMSO, or 1 μM of the indicated compounds and then counted after 48 h. Values were normalized to the count of control cells. **b-f** Mono Mac 6 cells were incubated in the presence or absence of 75 μg/ml CRP and the indicated concentrations of **b** Syk inhibitor, **c** SP600125, **d** okadaic acid, **e** LY294002, **e** wortmannin, or **g** sodium vanadate. Cells were counted after 48 h and counts were normalized to the no CRP/ no inhibitor or + CRP with no inhibitor values. Data were fit to sigmoidal dose–response curves with a variable Hill coefficient. **h** Mono Mac 6 cells were incubated as in A in the presence of 75 μg/ml CRP. All values are mean ± SEM, n = 4 independent experiments. * p < 0.05, **p < 0.01 (1-way ANOVA, Dunnett’s test)

**Fig. 8:**
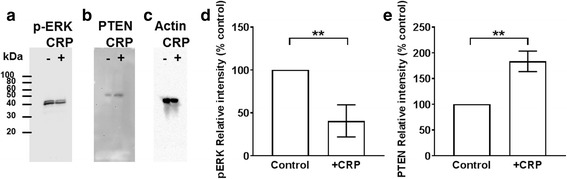
CRP regulates phospho-ERK and total PTEN levels. Mono Mac 6 cells were incubated in the presence or absence of 75 μg/ ml CRP for 24 h. Cells were then lysed and equal numbers of cells were analyzed by western blotting for **a)** phosphorylated ERK, **b)** PTEN, or **c)** actin as a loading control. Blots are representative of 3 independent experiments. The positions of molecular mass standards in kDa are at left. Staining for **d)** pERK at 24 h, and **e)** total PTEN at 24 h was quantified by densitometry. Values are mean ± SEM, n = 3 independent experiments. ** indicates p < 0.01 (t-test)

To elucidate the intracellular signaling pathways involved in the inhibition of proliferation by CRP, Mono Mac 6 cells were cultured with or without 75 μg/ ml CRP in the presence or absence of intracellular signaling inhibitors. At concentrations that did not inhibit cell proliferation in the absence of CRP, inhibitors of Src kinases (PP2), MEK1/2 (U0126), ras (peptide inhibitor), rac (NSC23766), Cdc42 (ML141), Syk (syk inhibitor), JNK (SP600125), or the serine/threonine protein phosphatases inhibitor okadaic acid did not significantly affect the ability of CRP to inhibit Mono Mac 6 proliferation (Fig. 7). However, the PI3 kinase inhibitors wortmannin and LY294002, and the tyrosine phosphatase inhibitor vanadate, increased the inhibitory effect of CRP on Mono Mac 6 proliferation, suggesting that PI3 kinase may block the CRP induced signal (Fig. 7d, e, f, and h). CRP regulates PI3K and ERK pathways in a variety of cell types [17, 48–51]. We found that CRP downregulated ERK phosphorylation, and upregulated the expression of PTEN, a phosphatase that regulates PI3K dependent PIP3 signaling (Fig. 8). These results suggest that the signal transduction pathway used by CRP to inhibit Mono Mac 6 proliferation is negatively regulated by PI3K.

### CRP inhibits proliferation and increases apoptosis of mono Mac 6 cells

**Fig. 9:**
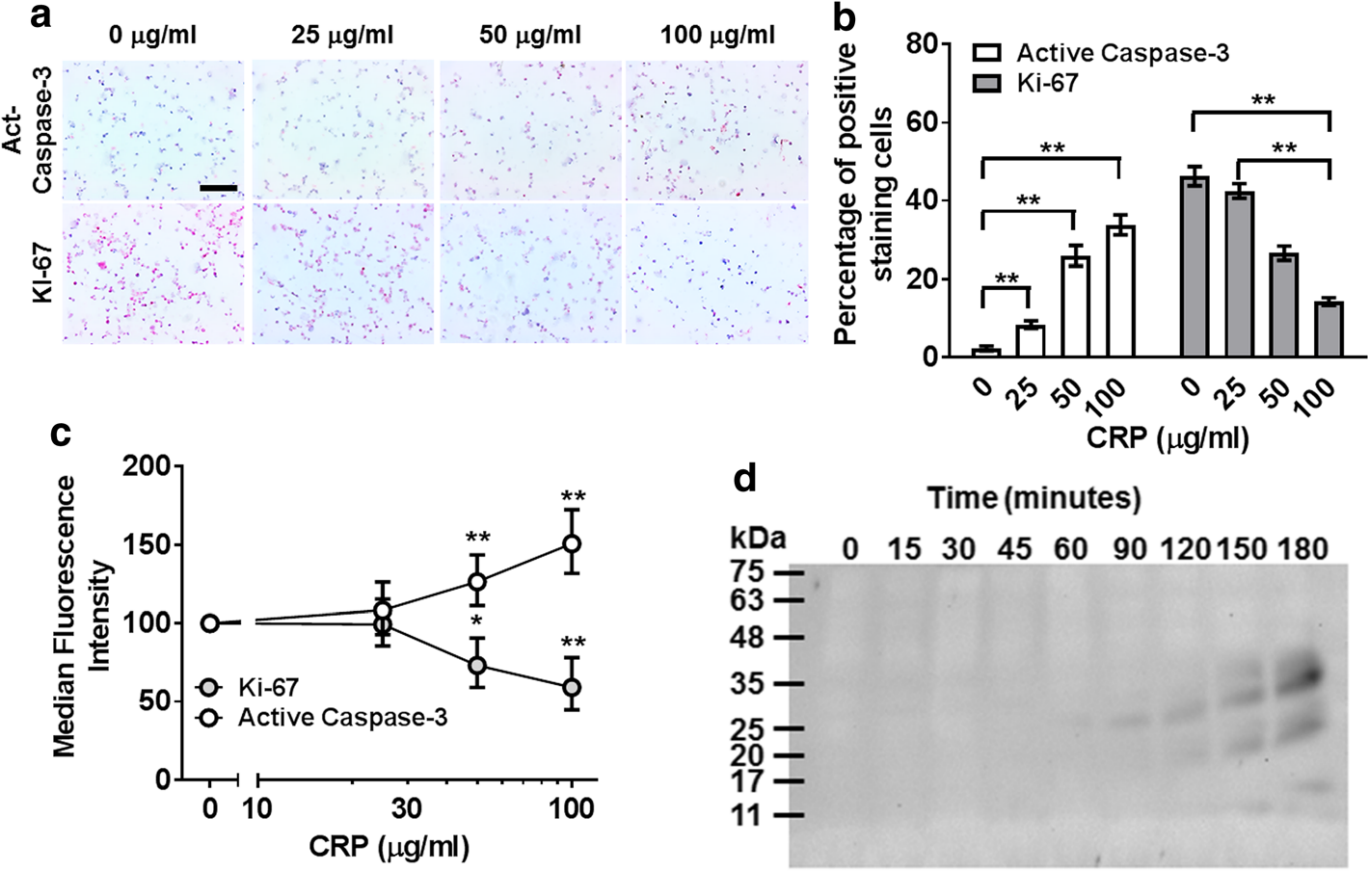
CRP inhibits the proliferation of and increases apoptosis of Mono Mac 6 cells. **a-c** Mono Mac 6 cells were incubated for 48 h in the presence or absence of the indicated concentrations of CRP. **a** Cells were then stained for active caspase 3 and Ki-67. Images are representative of 3 independent experiments. Bar is 100 μm. **b** Quantification of caspase 3 and Ki-67 staining. Values are mean ± SEM, n = 3 independent experiments. ** indicates *p* < 0.01 (t test)**. c** Flow cytometry analysis of Ki67 and active caspase 3 expression in Mono Mac 6 cells. Values are mean ± SEM, n = 3 independent experiments. * *p* < 0.05, ** *p* < 0.01 (t test). **d** Mono Mac 6 cells were incubated in the presence or absence of 75 μg/ml CRP for the indicated times. Cells were then lysed and western blots of the lysates were stained for active caspase 3. The position of molecular mass markers in kDa is at left. Image is representative of 3 independent experiments

Ki-67 is a cell proliferation marker, and caspase-3 is a key mediator of apoptosis [52, 53]. To test if CRP decreases the numbers of Mono Mac 6 cells by inhibiting proliferation and/or promoting apoptosis, we added CRP to Mono Mac 6 cells, and measured Ki-67 and cleaved caspase-3 levels. After 48 h, CRP increased cleaved caspase-3 levels and decreased Ki-67 levels (Fig. 9a-c). At 75 μg/ ml CRP, cleaved caspase 3 was detected within 3 h (Fig. 9d). These results suggest that CRP inhibits the proliferation of, and increases apoptosis of, Mono Mac 6 cells.

### CRP causes mono Mac 6 cells to accumulate in sub G1 phase

**Fig. 10:**
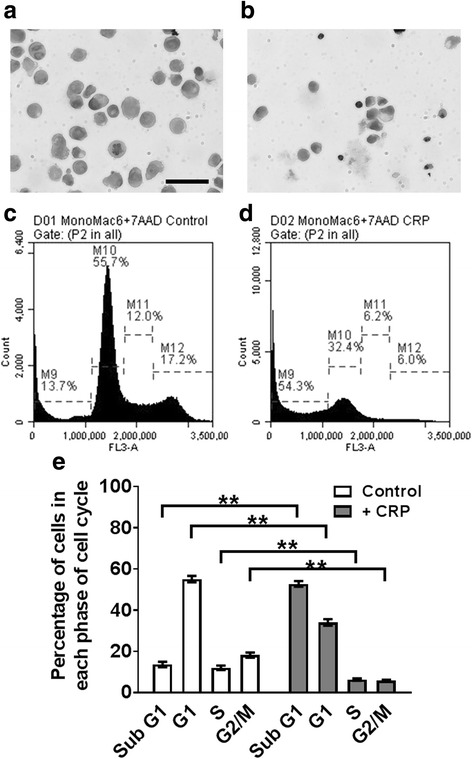
CRP induces cell cycle arrest in Mono Mac 6 cells. Mono Mac 6 cells were cultured with or without 75 μg/ ml CRP for 48 h. Cytospins of **a**) untreated and **b**) CRP-treated cells were stained with H&E. Images are representative of 3 independent experiments. Bar is 100 μm. **c** untreated and **d**) CRP-treated cells were stained with 7-AAD and staining intensity of cells was measured with a flow cytometer. Graphs are representative of 3 independent experiments. **e** Quantification of 7-AAD flow cytometry in Mono Mac 6. Values are mean ± SEM, n = 3 independent experiments. ** indicates *p* < 0.01 (t-test)

To elucidate how CRP slows Mono Mac 6 proliferation, we examined cells after 48 h of incubation with 75 μg/ml CRP. CRP caused many cells to be smaller than control cells (Fig. 10 a-b). CRP increased the percentage of cells in sub G1 phase (gate M9), and decreased the percentage of cells in G1 (gate M10), S (gate M11), and G2/M (gate M12) phases. These results suggest that CRP inhibits cell cycle progression and induces DNA fragmentation in Mono Mac 6 cells.

## Discussion

Pentraxins are involved in regulating many aspects of the innate immune system, such as adhesion, differentiation, and polarization of monocyte/macrophages. In this report, we observed that CRP, but not SAP or PTX3, inhibited the proliferation of, and induced apoptosis of, the acute myeloid leukemia (AML) cell line Mono Mac 6. These data suggest that CRP might regulate the proliferation of some AML cells.

We found that the IC50 of CRP on Mono Mac 6 is 75 μg /ml. In healthy humans, the plasma levels of CRP are low (< 2 μg/ml), whereas during inflammation, CRP levels may rise to 1000 μg/ml [2, 54]. Elevated plasma levels of CRP are not only associated with a wide variety of inflammatory diseases but also some forms of cancer. Although CRP is not thought to be directly associated with AML and chronic myeloid leukemia (CML), complications associated with leukemia, such as infections, and elevated systemic cytokines, such as IL-6, lead to elevated CRP levels [55–57]. Intriguingly, low levels of CRP after stem cell transplantation therapy for AML and CML are an independent variable predicting the risk of a relapse after stem cell transplantation [58], and conversely high levels of CRP may correlate with recovery [59].

Pentraxins bind to multiple receptors including IgG Fcγ receptors, the IgA receptor FcαR, selectins, and DC-SIGN [5, 17–21]. CRP shows high affinity binding to FcγRI, FcγRII, and FcαR [17, 48, 60]. Anti- FcγRI (CD64) antibodies were able to partially block the ability of CRP to inhibit Mono Mac 6 proliferation, suggesting that FcγRI might mediate this effect of CRP. However, we were unable to replicate the effects of CRP on Mono Mac 6 cells with any combination of antibodies to FcγRI, FcγRII, and FcαR, suggesting that the interaction of CRP with FcγRI, FcγRII, and FcαR is fundamentally different than the interaction of the antibodies we used with these receptors, or that CRP binds to unknown receptors found on Mono Mac 6 that mediate or co-mediate the CRP effect on Mono Mac 6 proliferation.

Although we observed that CRP inhibits the proliferation and induces apoptosis of an AML cell line, others have noted that CRP can promote cell survival in a variety of cell types. CRP promotes myeloma cell survival via a CD32-dependent pathway involving PI3 kinase, ERK, and NF-κB [48], and CRP can stimulate the proliferation of U266 multiple myeloma cells by up-regulating the expression of survivin and HSP90α proteins [61]. CRP also promotes the proliferation of human pulmonary artery smooth muscle cells via PI3 kinase, ERK, and NF-κB pathways [50]. CRP also promotes the survival of podocytes (epithelial cells in the Bowman’s capsule of the kidney) by a PI3 kinase-dependent pathway [62]. Conversely, in cardiac myocytes, osteoclasts, and endothelial cells, CRP induces PI3 kinase, ERK, and PTEN pathways to inhibit cell differentiation, proliferation, and survival [63–65]. These data and our data suggest that CRP can either induce or inhibit cell proliferation and survival depending on the cell type.

## Conclusion

Together, these results suggest that concentrations of CRP that can occur during inflammation inhibit the proliferation and induce apoptosis of at least one AML cell line, and that elevating levels of CRP or treatment with a CRP mimetic may be possible therapies for some forms of AML.

## Abbreviations

FcγR: Fcγ receptors
FcγR; CD89: IgA receptor
AML: Acute myeloid leukemia
BCS: Bovine calf serum
CD62L: L-selectin
CD62P: P-selectin
CML: Chronic myeloid leukemia
CRP: C-reactive protein
DC-SIGN: Dendritic cell-specific intercellular adhesion molecule-3-grabbing non-integrin
PBMC: Peripheral blood mononuclear cells
PI3K: Phosphatidylinositol −3-kinase
PTX3: Pentraxin-3
SAP: Serum amyloid P
